# Supplementary material for: Age-Stage, Two-Sex Life Table of Leptinotarsa decemlineata (Coleoptera: Chrysomelidae) Experiencing Cadmium Stress
Source: Insects. 2025 Jan 13;16(1):73. doi: 10.3390/insects16010073 (PMC11765768; doi:10.3390/insects16010073)

Figure S1

Age-Stage, Two-Sex Life Table of *Leptinotarsa decemlineata* (Coleoptera: Chrysomelidae) Experiencing Cadmium Stress

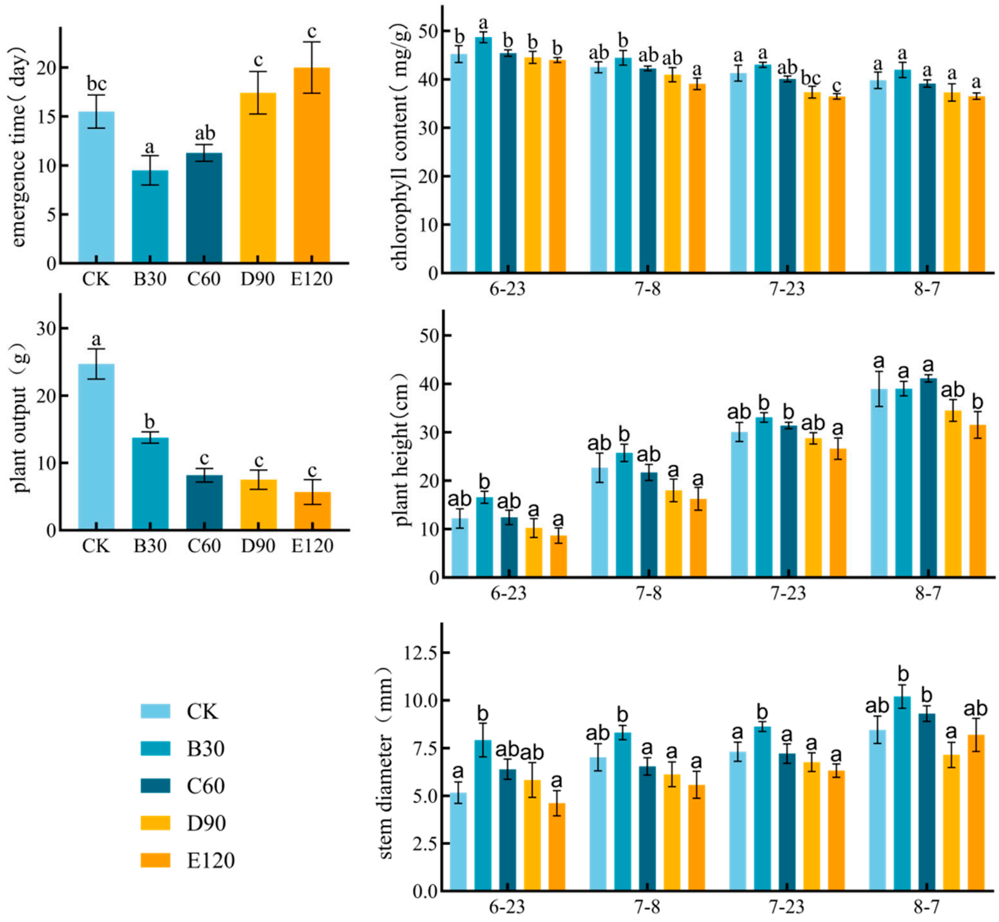

Supplement: Supplementary file 1 [file insects-16-00073-s001.zip › insects-3383265-supplementary.pdf]
